# Harnessing iChip technology for novel antibiotic discovery from peat soil microbiomes to combat antimicrobial resistance

**DOI:** 10.3389/fmicb.2025.1530273

**Published:** 2025-02-21

**Authors:** Lutfi Chabib, Tedi Rustandi, Muhammad Hafizh Abiyyu Fathin Fawwazi, Eka Kumalasari, Desy Ayu Lestari, Senya Puteri Amalia, Normilawati Normilawati

**Affiliations:** ^1^Department of Pharmacy, Faculty of Mathematics and Natural Sciences, Universitas Islam Indonesia, Yogyakarta, Indonesia; ^2^Department of Pharmacy, Sekolah Tinggi Ilmu Kesehatan Insan Sarjana Farmasi Indonesia (ISFI) Banjarmasin, Banjarmasin, Indonesia; ^3^Department of Chemistry, Faculty of Mathematics and Natural Sciences, Lambung Mangkurat University, Banjarbaru, Indonesia; ^4^Prodi Magister Kimia, Fakultas Matematika dan Ilmu Pengetahuan Alam (MIPA) Universitas Lambung Mangkurat, Banjarbaru, Indonesia

**Keywords:** antimicrobial resistance, microorganisms, peatlands, microbial cultivation techniques, co-culture, *in situ* culturing, uncultured microorganisms, environmental microbes

## 1 Introduction

Antimicrobial resistance (AMR) poses a critical global health threat, complicating infection management worldwide. Data on the prevalence of antibiotic resistance released by the World Health Organization (WHO) in 2019 has caused the deaths of 1.27 million people (Murray et al., 2022; WHO, 2023). Additionally, the World Bank estimates that the economic impact of AMR could reach a loss of up to US$ 1 trillion in healthcare costs by 2050 and a gross domestic product (GDP) loss of US$ 3.4 trillion by 2030 (Jonas et al., 2017).

The urgent need to discover new drugs to replace resistant antibiotics has become increasingly critical. One of the largest sources of new antibiotic producers comes from the soil, which harbors 99% of microbial species. Antimicrobial compounds are produced by microbes in soil that often remain unculturable in the laboratory due to the limitations of traditional cultivation techniques, which fail to replicate the microbes' natural habitats (Choi et al., 2015; Bhattacharjee, 2022). The type of soil that has great potential for obtaining new antibiotic agents is peat soil (Kujala et al., 2018; Liu et al., 2022; Atapattu et al., 2023). Peat soil contains organic deposits rich in nutrients that support microbial growth and diversity (Nawan and Wasito, 2020).

The abundant microbial content in peat soil needs to be effectively harnessed to develop new antibiotics. Current microbial cultivation techniques are generally limited to only a subset of microbes, restricting the isolation of secondary metabolites. Overcoming these limitations requires innovative approaches to cultivate antibiotic-producing microbes that remain unculturable under laboratory conditions. Uncultured Soil Technology (UST) or *in situ* incubation is one of the latest developments, which involves cultivation using natural growth factors present in the environment (Berdy et al., 2017; Chaudhary et al., 2019).

A prominent *in situ* incubation method is the isolation chip (iChip) technique, developed by D. Nichols in 2010, which led to his first discovery. This method enabled the discovery of Teixobactin, a groundbreaking antibiotic (Berdy et al., 2017; Zhao et al., 2023). This article reviews several relevant articles published between 2015 and 2024 to address the question, “How is the discovery of new antibiotics from peat soil using the iChip method?” This Opinion Article aims to provide a critical discussion, highlighting the opportunities and challenges in harnessing the potential application of iChip technology for the discovery of novel antibiotics from peat soil microbiomes. This article is intended to encourage original experimental studies focusing on peat soil, which holds significant potential.

## 2 Sample preparation

### 2.1 Sample collection

Peatlands, which encompass the most extensive total area in the world, are predominantly found on the Asian continent, accounting for 38.4% of the world's peatlands. The regions in Asia with the largest peatland areas are Asian Russia, Indonesia, and Malaysia. Following Asia, other regions that have large amounts of peatland after the Asian zone are North America (31.6%), Europe (12.5%), South America (11.5%), Africa (4.4%), and Australasia and Oceania (1.6%) (Xu et al., 2018).

Previous studies have discussed the optimal sampling depth for producing microbes. Sampling in research using the culture isolation method taken at 0 to 15 cm depth can isolate *Actinomycetes* microbes (Atapattu et al., 2023). Samples taken at a depth of 50 cm produced *Enterobacteriaceae* (46.4%), *Bacillaceae* (28.6%), *Streptococcaceae* (10.7%), *Staphylococcaceae* (10.7%), and *Clostridiaceae* (3.6%) microbes (Mahdiyah et al., 2020).

Peat soil characteristics, such as acidic pH (3.5 to 4.1) (Nawan and Wasito, 2020; Goh et al., 2022; Atapattu et al., 2023), temperature (tropical climate with an average temperature of 28°C), nutrient availability, and substrate composition, influence microbial abundance and diversity, are located in lowlands, and are inundated due to excessive rainfall (average rainfall 200 cm^3^ per year) (Nawan and Wasito, 2020; Paul et al., 2021; Goh et al., 2022). In addition, peat soil has waterlogged conditions and low nutrient availability, which influence the characteristics of the secondary metabolites produced (Weeraphan et al., 2023).

### 2.2 Sample preparation

Peat soil samples were collected from a depth of 0–50 cm. Sampling was conducted beneath trees, with an emphasis on root systems to enhance microbial diversity. When feasible, the *in situ* incubation process was initiated directly at the sampling site. If sample transportation to another location was required, the samples were preserved at 4°C in darkness until the *in situ* incubation process could proceed. This cooling step is essential for maintaining sample integrity by preserving their chemical and biological properties and preventing alterations caused by external factors such as light, elevated temperatures, or desiccation. This methodology aims to create a controlled environment that replicates the microbes' natural habitat, thereby promoting their survival and metabolic activity during incubation (Preston and Basiliko, 2016; Ong et al., 2020; Tate, 2021; Goh et al., 2022; Polrot et al., 2022).

## 3 Isolation chip (iChip) technology for isolation peat soil

### 3.1 Environmental condition: collection and preparation

The sample underwent serial dilutions before being intervened in an agar medium. The agar medium that can be used is Tryptic Soy Agar (TSA). Agar plates were then incubated for 3–5 days at room temperature. After incubation, the number of colonies was counted to determine the optimal dilution amount before inoculating a single bacterial cell into the diffusion chamber (Polrot et al., 2022). The minimum value of CFU/g peat soil in previous studies was log 5.7, and the maximum value was 8.0, so it can be used as a reference in carrying out serial dilutions (Ramata-Stunda et al., 2015; Glushakova et al., 2021). This step is carried out in the laboratory to determine the abundance of bacteria outside their natural environmental conditions.

The iChip design comprises several parts developed by Berdy et al. (2017). The parts of the iChip include (1) a middle plate containing through-holes, (2) semipermeable membranes affixed to both the front and back of the middle plate to separate the plate from the environment, and (3) a membrane seal designed as a panel board, to reinforce the structure (Berdy et al., 2017).

All iChip components must be sterilized prior to assembly and use in the incubation process. Sterilization is performed by soaking the components in 70% ethanol for 15 min, followed by air drying at room temperature until completely dry. Additionally, a sediment bucket filled with peat soil is prepared for the incubation of the iChip device (Polrot et al., 2022).

### 3.2 *In situ* incubation: iChip

The iChip technology consists of hundreds of diffusion chambers containing an agarose medium or other media, such as molten SMS medium (0.125 g casein, 0.1 g potato starch, 1 g casamino acids, 20 g bacto-agar, dissolved in 1 liter of water). This medium is designed to support bacterial growth during the incubation process for Grasses and sediment samples, which can be applied to peat soil samples, but further experiments are needed (Ling et al., 2015; Polrot et al., 2022). Another medium that can be used and has been proven in previous studies for peat soil samples is TSA, with a concentration of 10% (according to the acidic conditions of peat soil) (Liu H. et al., 2021; Goh et al., 2022).

The initial step of *in situ* incubation involves immersing the iChip device in the prepared medium, which has been supplemented with samples at the optimal concentration, as determined in Section 3.1. This procedure ensures the sample's distribution into the through-holes on the iChip plate. After immersion, the agarose medium containing the sample is allowed to solidify.

Subsequently, the diffusion chamber is sealed by placing a plate directly over the through-holes and applying petroleum jelly around the edges to secure the sample's position within the chambers during the incubation period. Once prepared, the iChip device is wrapped with a parafilm protector and placed in a sediment bucket containing peat soil to begin the incubation process. During incubation, the diffusion chambers are positioned as close as possible to their natural environmental conditions. This setup is achieved by separating the sample from the external environment using a semipermeable membrane, which allows for nutrient and waste exchange while maintaining microbial viability. The incubation process lasts for 7 days (Polrot et al., 2022).

### 3.3 Isolation of secondary metabolite screening for activity

The iChip device that has completed the incubation process is continued to the microbial isolation stage. The iChip is rinsed with sterile distilled water and subsequently incubated at 20°C for several weeks under room temperature conditions and in the dark (Polrot et al., 2022). The grown isolates were cultured in seed broth containing 15 g glucose, 10 g malt extract, 10 g soluble starch, 2.5 g yeast extract, 5 g casamino acids, and 0.2 g CaCl_2_•2H_2_O per 1 liter of deionized H_2_O with pH adjustment of 7.0. The cultures were diluted 1:20 into four different types of fermentation media (Ling et al., 2015; Quigley et al., 2020).

The fermentation process is carried out over 11 days at 29°C with continuous stirring. Following fermentation, the culture was dried and dissolved in 100% dimethyl sulfoxide (DMSO). The resulting extract was tested for antimicrobial activity against *Staphylococcus aureus* using Mueller-Hinton Agar (MHA) plates, which are incubated for 20 h at 37°C. Antimicrobial activity was assessed by observing the presence of a clear inhibition zone around the test area (Ling et al., 2015).

The fermentation and purification processes are based on the methodology for producing the secondary metabolite teixobactin, as outlined by Ling et al. (2015). Large-scale production employs the Sartorius Biostat Cultibag STR 50/200 bioreactor. Subsequently, extraction and purification are conducted using specific solvents and methods tailored to the metabolite being isolated (Ling et al., 2015).

## 4 Secondary metabolite

Secondary metabolites produced by the genus *Streptomyces* obtained from peat soil of the Nong Jum Rung peat swamp forest (Rayong Province, Thailand) include geldanamycin, 17-O-demethylgeldanamycin, reblastatin, 17-demethoxyreblastatin, nocardamine, and dehydroxynocardamine (Weeraphan et al., 2023). Secondary metabolites produced using *in situ* incubation methods and similar methods can be seen in Table 1.

**Table 1:** The discovery of new antibiotics using *in situ* incubation soil technology.

**Antibiotics**	**Bacteria/Isolate**	**Sample**	**Isolation method of producing strains**	**References**
Teixobactin	*E. terrae ssp*. Carolina	Grassy field in Maine	1. Growth Media: SMS media (0.125 g casein, 0.1 g potato starch, 1 g casamino acids, 20 g bacto-agar in 1 liter of water) 2. iChip Design: The structure of iChip consists of 3 parts, including a middle plate, a semipermeable membrane, and two side panels. The place where microorganisms grow is on the middle plate, which consists of holes blocked by a semipermeable membrane from the outside environment. The two side panels function as structural supports.	Ling et al., 2015
Streptomycobactin, kitamycobactin, and amycobactin	*Streptomyces sp* (Streptomycobactin), *Kitasatospora sp*. (kitamycobactin), *Amycolatopsis sp* (Amycobacti)	Location and type of soil are not explicitly stated		Quigley et al., 2020
Clovibactin	*E. terrae ssp*. Carolina	Isolate P9846 (clovabactin producer) originating from sandy soil in North Carolina.		Shukla et al., 2023
–	107 bacterial strains in 17 genera, including *Lysobacter sp*. and *Alkalihalobacillus strains*	Hot water samples (90°C) from the DaGunGuo in Rehai, Tengchong, Yunnan, China	1. Using the modified ichip method to cultivate heat-tolerant microorganisms. 2. Growth Media: gellan gum 3. iChip Design: The material used is polypropylene plastic with a thickness of 5 mm, a diameter of 5 cm, and an inner diameter of 3 cm. There are 37 holes that function to incubate microbes, which have a diameter of 3 mm for each hole and a distance between holes of 4.5 mm. The PCTE membrane (pore size 0.03 μm) is attached to the center plate using heat-resistant glue (RTV 108 glue).	Zhao et al., 2023
–	*Oceanisphaera sp., Pseudomonas sp., Bacillus sp*	Liverpool Dock sediment	1. Growth Media: Agarose medium 2. iChip Design: The device has two arrays of 192 through-holes with a membrane that interfaces with the environment. The membrane is a polycarbonate composition measuring 27 cm in diameter with a pore size of 0.03 μm.	Polrot et al., 2022
–	*Proteobacteria, Actinobacteria, Bacteroidetes, Firmicutes, Verrucomicrobia*	Intertidal sediment and near-shore seawater were collected from Charlottetown Harbor.	1. The method used is microencapsulation followed by *in situ* incubation 2. Growth Media: Agarose medium 3. *In situ* Design: Prepared bacteria are inserted into an agarose solution, and the mixture is then inserted into a microfluidic microchip to form microbeads with a diameter of 80 ± 20 μm. The incubation process uses Slide-A-Lyzer™ gamma-irradiated dialysis cassettes (Thermo Fisher, Canada).	Pope et al., 2022
–	*Lapillicoccus, Flavitalea, Quadrisphaera, Motilibacter, and Polymorphobacter*	Soil in Hennequin Point, Admiralty Bay, King George Island, Antarctica	1. Growth Media: 1/100 diluted (0.08 g per liter) Difco Nutrient Broth – NB (Becton Dickinson, United States) plates (1/100 NB), solidified using 0.7% (w/v) Gellan gum (CP Kelco, United States) 2. *In situ* Design: Plates were incubated in polyethene bags to prevent drying.	Pulschen et al., 2017
–	*Alphaproteobacteria, Actinobacteria, Bacteroidetes, Firmicutes, Gammaproteobacteria* (only from PS medium), *Acidobacteria* (only from PS medium)	Originating from two types of environments at Hokkaido University, Sapporo, Japan. Forest Soil and Pond Sediment.	1. Growth Media: PYG Medium 2. (Composition: Peptone: 0.1 g/L, Yeast Extract: 0.1 g/L, Glucose: 0.1 g/L, Phosphate Buffer (20 mM, pH 7): For pH stabilization, Agar (15 g/L): as a solidifying agent). 3. This medium was applied using two methods: a. PT Medium (Phosphate and Agar Together) b. PS Medium (Phosphate and Agar Separately) 4. *In situ* Design: Forest Soil: Collected from a depth of 5–10 cm in deciduous forest. Pond Sediment: Collected from the 0–10 cm layer of the pond bottom. The samples were then suspended in sterile saline solution (0.9% NaCl), serially diluted, and inoculated onto PT and PS media. The media were incubated at 25°C in the dark for up to 3 weeks.	Kato et al., 2018
–	*Alpha-proteobacteria Beta-proteobacteria, Gamma-proteobacteria, Actinomycetes, Acidimicrobiia, Thermoleo-philia, Flavobacteriia, Cytophagia, Opitutae Balneolia, and Cyanophyceae*	Sediments from the South China Sea (S1) and the Mariana Trench (S2) were collected in 2019 and 2021.	1. Growth Media: Modified nutrient media: a. 0.5% alkali-lignin (Lig medium) b. 0.5% starch (St medium) c. Artificial seawater (ASW medium) 2. *In situ* Design: The samples were placed into a “microbial aquarium,” followed by enrichment in Lig and St media. Dilution and inoculation were carried out, and identification was performed using PCR.	Ahmad et al., 2024
–	*Actinobacteria, Bacteroidetes, Chloroflexi, Firmicutes, and Proteobacteria*	Three types of sediment samples were collected from the intertidal zone on the northwest coast of Meishan Island (Ningbo, China) between June and September 2019.	1. Growth Media: a. 1:2 diluted seawater b. 1:10 diluted liquid R2A medium c. 1:100 diluted nutrient broth 2. All media were supplemented with artificial seawater salt to a final concentration of 2%. 3. *In situ* Design: Diffusion Chamber (DC); Dilution-to-Extinction (DTE); Preparation Step (PS media)	Jung et al., 2021

## 5 Comparative analysis in microbial isolation

**Figure 1:**
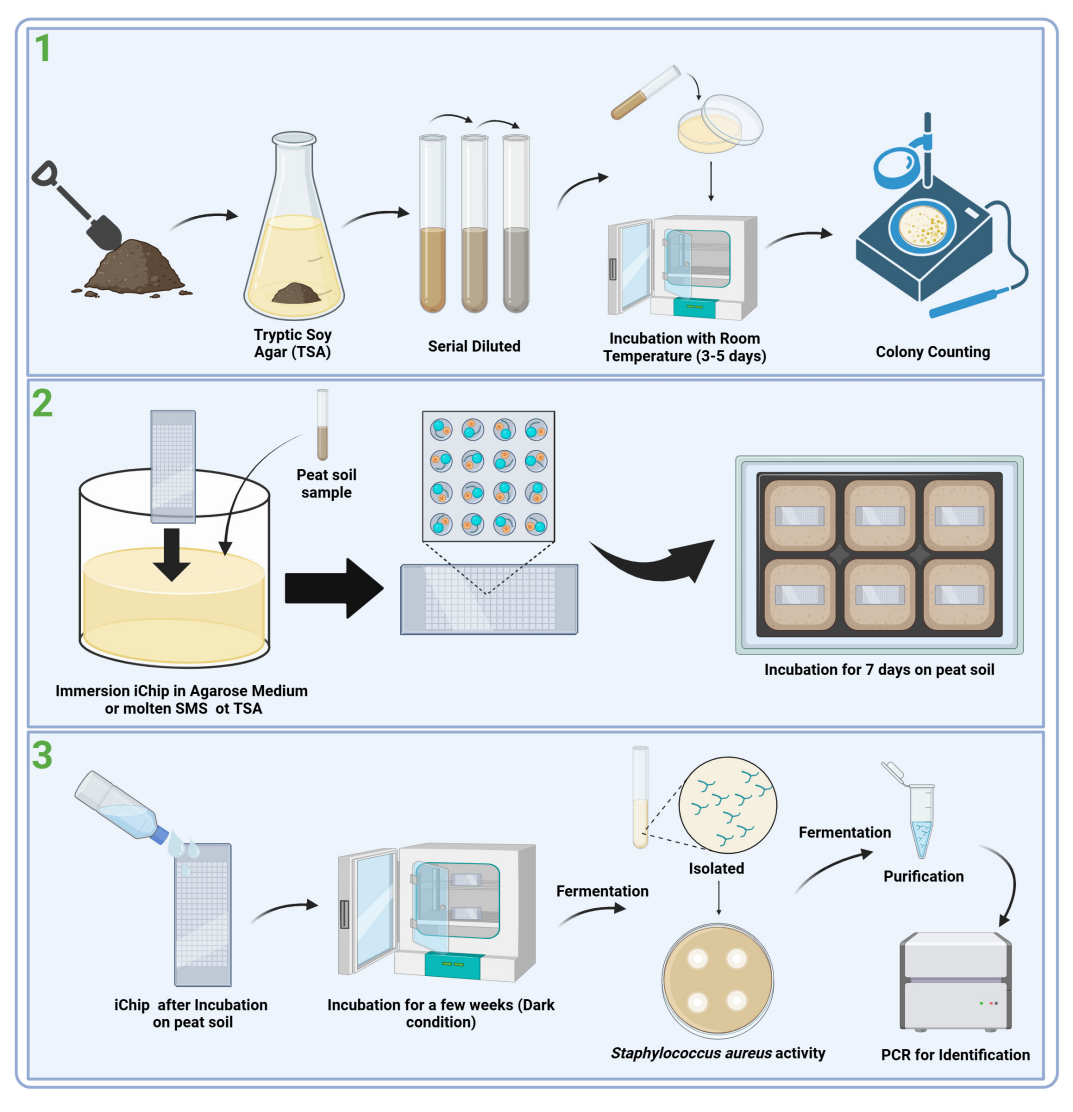
Workflow for the discovery of novel antibiotic agents from peat soil (Modolon et al., 2023; Zhang and Zhang, 2023; Ahmad et al., 2024). (1) Environmental condition: Collection and preparation, (2) *In situ* incubation: iChip, and (3) Isolation of secondary metabolite screening for activity.

The application of iChip technology in studies involving marine water columns and soil microorganisms has demonstrated superior results compared to traditional cultivation methods using standard Petri dishes. Comparison of tests resulted in several findings, including (1) Increased microbial recovery: the recovery of colonies was five times higher with the iChip method, with 40%−50% of cells incubated in iChips forming microcolonies or 5 to 300 times more compared to traditional methods (Berdy et al., 2017), (2) access to novel microorganisms and reduced cultivation bias: the iChip method yielded a much higher level of phylogenetic uniqueness, increasing the richness and uniformity of isolates that are representative microorganisms of the microbial community is biased (Berdy et al., 2017; Lodhi et al., 2018; Liu X. et al., 2021; Liu H. et al., 2021), and (3) the iChip has been shown to produce new antibiotics such as Teixobactin and Clovabactin, which is produced by a previously unknown soil bacteria (tentatively named *Eleftheria terrae*), and N-Acyltyrosine from *Alteromonas sp*. (Ling et al., 2015; Sherpa et al., 2015; MacIntyre et al., 2019; Shukla et al., 2023). The flow of the *in situ* incubation method using iChip on peat soil can be seen in Figure 1.

## 6 Conclusion

The *in situ* incubation method represents an innovative approach to discovering new antibiotic agents to address the growing issue of drug resistance. One notable application of this method is the iChip technology, which has been employed across various soil types. Among these, peat soil stands out as a highly promising source of secondary metabolites due to the rich microbial diversity it harbors, yet it remains largely unexplored.
